# Is perfect good? – Dimensions of perfectionism in newly admitted medical students

**DOI:** 10.1186/s12909-017-1034-9

**Published:** 2017-11-13

**Authors:** Helen Seeliger, Sigrid Harendza

**Affiliations:** 1 III. Department of Internal Medicine, University Medical Center Hamburg-Eppendorf, Hamburg, Germany; 2 Universitätsklinikum Hamburg-Eppendorf, III. Medizinische Klinik, Martinistr. 52, D-20246 Hamburg, Germany

**Keywords:** Anxiety, Big five, Depression, Medical school admission, Perfectionism, Undergraduate medical education

## Abstract

**Background:**

Society expects physicians to perform perfectly but high levels of perfectionism are associated with symptoms of distress in medical students. This study investigated whether medical students admitted to medical school by different selection criteria differ in the occurrence of perfectionism.

**Methods:**

Newly enrolled undergraduate medical students (*n* = 358) filled out the following instruments: Multidimensional Perfectionism Scale (MPS-H), Multidimensional Perfectionism Scale (MPS-F), Big Five Inventory (BFI-10), General Self-Efficacy Scale (GSE), Patient Health Questionnaire 9 (PHQ-9), and Generalized Anxiety Disorder 7 (GAD-7). Sociodemographic data such as age, gender, high school degrees, and the way of admission to medical school were also included in the questionnaire.

**Results:**

The 298 participating students had significantly lower scores in Socially-Prescribed Perfectionism than the general population independently of their way of admission to medical school. Students who were selected for medical school by their high school degree showed the highest score for Adaptive Perfectionism. Maladaptive Perfectionism was the strongest predictor for the occurrence symptoms of depression and anxiety regardless of the way of admission.

**Conclusions:**

Students from all admission groups should be observed longitudinally for performance and to assess whether perfectionism questionnaires might be an additional useful instrument for medical school admission processes.

## Background

Perfectionism is a multidimensional concept which includes the striving for flawlessness and for setting high goals [1, 2]. Society expects its doctors to be flawless and to do their job as good as possible [3]. Rewarding perfectionism as an important personality trait in physicians has become a concept in the health care system in the late 90s of the twentieth century [4]. Even medical students already show higher personal standards compared to arts students [5]. On the other hand, a medical culture that emphasizes perfectionism, denial of personal vulnerability, and delayed gratification has deleterious effects on physicians’ wellbeing leading to suboptimal care and medical errors [6]. Meaningful levels of stress and a high rate of psychiatric symptoms are already present in medical students [7] and higher scores in perfectionism dimensions, i.e. severe perfectionism, are associated with psychiatric levels of distress in medical students [8]. Working as perfectly as possible without becoming seriously distressed seems to be the path medical students need to find. For medical educators, the challenge seems to be in selecting the type of students who will be able to keep this balance in order to become good doctors.

How to select the right medical student is an ongoing debate [9]. Many different selection formats are being used worldwide, e.g. high school grade point average [10, 11], emotional intelligence tests [12], interviews [13], multiple mini-interviews [14], situational judgement tests [15], lottery [16, 17], and others. With respect to exam results, the high school grade point average seems to predict the future success at medical school and in postgraduate training best [18]. In Germany, applicants for undergraduate medical studies have to send a list of their six favorite medial schools to a central office. A selection process chosen by each medical school individually admits 60% of the students who named this school as their primary choice, 20% are admitted by their high school grade point average, and 20% by other means (e.g. a waiting period – students with lower grade point averages can raise their grade point averages by a certain amount per year that they wait; the average waiting period is approximately six to seven years). The individual selection process at the Medical Faculty of the University of Hamburg consists of two steps: according to the law, students have to be invited by grade point average to participate in a natural sciences test (HAM-Nat) [19] when they have selected Hamburg as their first choice and their grade point average is too low to be admitted by grade point average alone. Students who are not directly admitted by the HAM-Nat but receive high scores in this test are invited to participate in multiple mini-interviews (HAM-Int) [20].

Students with formidable high school grade point averages have already proven that they are striving for high achievements. Since it has been shown that different medical school selection processes call upon different personality characteristics [21] and given the association of severe perfectionism as a personality trait with an increased risk for anxiety and depressive disorders in medical students [8] we asked the research question whether medical students admitted by different selection criteria differ in their perfectionism scores.

## Methods

### Study design

In October 2016, we invited all incoming 358 first-year undergraduate medical students newly enrolled at the Medical Faculty of the University of Hamburg to participate in this study during their orientation week before the start of the first semester. The students were approached by their tutors during the orientation week. They participated voluntarily in this study and filled out a questionnaire including questions about their sociodemographic data, their high school degrees, and their way of admission to medical school. Furthermore, the questionnaire included the following instruments: Multidimensional Perfectionism Scale by Hewitt and Flett (MPS-H) [2], Multidimensional Perfectionism Scale by Frost (MPS-F) [1], Big Five Inventory 10 (BFI-10) [22], General Self-Efficacy Scale (GSE) [23], Patient Health Questionnaire 9 (PHQ-9) [24], and Generalized Anxiety Disorder 7 (GAD-7) [25]. The Ethics Committee of the Hamburg Chamber of Physicians approved this study (WF-047/16) and students gave their consent for participation. All questionnaires contained anonymized codes only decipherable by the students. Completion of the questionnaires took approximately 15 min.

### Instruments

#### Multidimensional perfectionism scale (MPS-H)

This validated instrument [2, 26, 27] includes 45 items and measures the following three dimensions with 15 items each: Self-Oriented Perfectionism (SOP) (e.g. ‘I strive to be the best at everything I do’), Other-Oriented Perfectionism (OOP) (e.g. ‘If I ask someone to do something, I expect it to be done flawlessly’), and Socially-Prescribed Perfectionism (SPP) (e. g. ‘The people around me expect me to succeed at everything I do’). All items are rated on a 7-point Likert scale (1 = disagree; 7 = agree). Normative data are available for the MPS-H.

#### Multidimensional perfectionism scale (MPS-F)

This validated instrument [1] includes 35 items and measures the following six dimensions: Concern over Mistakes (CM) (nine items) e. g. ‘I should be upset if I make a mistake’, Personal Standards (PS) (seven items) e. g: ‘I set higher goals than most people’, Parental Expectations (PE) (five items) e.g. ‘My parents expected excellence from me’, Parental Criticism (PC) (four items) e.g. ‘I never felt like I could meet my parents’ expectation’, Doubts about Action (DA) (four items) e.g. ‘Even when I do something very carefully, I often feel that it is not quite right’, and Organization (O) (six items) e.g. ‘I am an organized person’. In the German version [28], the items are rated on a Likert scale from 1 to 6 (1 = not at all true, 2 = not true, 3 = moderately not true, 4 = moderately true, 5 = true, 6 = exactly true). No normative data are available for the MPS-F.

#### Big five inventory (BFI-10)

The BFI-10 [22, 29] is a short instrument to assess the five dimensions of the five-factor model extraversion, agreeableness, conscientiousness, neuroticism and openness. Each dimension measures by means of two items, how well the statements describe the personality of oneself, on a 5-point Likert scale (1 = disagree strongly, 2 = disagree a little, 3 = neither agree or disagree, 4 = agree a little, 5 = agree strongly). Sample items for the BFI-10 are: I see myself as someone who …: 1) is reserved, 2) tends to be lazy, 3) gets nervous easily, 4) has an active imagination [22].

#### General self-efficacy scale (GSE)

The GSE [23, 30] is a one-dimensional scale for the measurement of a general sense of perceived self-efficacy. It contains 10 items (e.g: ‘It is easy for me to stick to my aims and accomplish my goals’) on a 4-point Likert scale (1 = not at all true, 2 = hardly true, 3 = moderately true, 4 = exactly true).

#### Patient health questionnaire 9 (PHQ-9)

The PHQ-9 [24, 31] is an instrument to assess depression. It contains nine questions about depressive symptoms, such as feeling tired or having little energy, and their occurrence over the last 2 weeks. They are rated on a 4-point scale (0 = not at all, 1 = several days, 2 = more than half the days, 3 = nearly every day). A score ≥ 10 provides good sensitivity and specificity for a major depression [24].

#### Generalized anxiety disorder 7 (GAD-7)

This instrument [25, 32] assesses generalized anxiety disorder with seven items about the occurrence of symptoms over the last 2 weeks e.g. feeling nervous, anxious or on edge or being able to stop or control worrying which are rated on a 4-point scale (0 = not at all, 1 = several days, 2 = more than half the days, 3 = nearly every day). A score ≥ 10 represents a reasonable cut off for identifying GAD [25].

### Statistical analyses

If at least 80% of the items were filled out per questionnaire, we included the questionnaire in the data set and the missing data were replaced by the mean of the scale. The missing number of items were as follows: MPS-F: 54 items of 10,430, MPS-H: 153 items of 13,410, BFI-10: 10 items of 2980, PHQ-9: 21 items of 2682, GAD-7: 13 items of 2086, GSE: 17 items of 2980. If the measures had existing normative data, we transformed the raw data into t-scores. If the age was missing, we used the mean of our sample to transform the data into t-scores. If the gender was missing, we did not transform the raw data (one case). Composite measures were created of Z-transformed scores of the subscales SOP from the MPS-H and PS from the MPS-F for Adaptive Perfectionism (AP) as well as of the subscales SPP from the MPS-H and CM as well as DA from the MPS-F for Maladaptive Perfectionism (MP) [5]. The data were analyzed using IBM SPSS statistics version 23. The Cronbach’s alpha for the different instruments was as follows: MPS-F: Personal standards: .77, Organization: .90, Concern over mistakes: .86, High parental expectations: .82, Parental criticism: .75, Doubt about action: .64; MPS-H: SOP: .89, OOP: .80, SPP: .87. GSE: .80. PHQ-9: .77. GAD-7: .80. BFI-10: Neuroticism: .51, Extraversion: .77, Openness: .57, Agreeableness: .28, Conscientiousness: .46. To explore group differences, we used single analysis of variance and Bonferroni post hoc comparisons. The reported MPS-H data are based on standardized t-scores to permit comparison of the result with the normal population. A stepwise regression was calculated to investigate variables, which predict the PHQ-9 and GAD-7 scores of the students. Variables with a significance level of *p* < .05 were included.

## Results

Of the 358 enrolled undergraduate medical students, 298 (189 female, 108 male) filled in the questionnaire completely (response rate 83.2%). Table 1 shows their sociodemographic data. Fifty participants gained entrance to Hamburg Medical School by their high school degree, 98 by their result in a natural sciences test (HAM-Nat), 66 students by multiple mini interviews (HAM-Int), and 51 students by a waiting period after application. A small group of 33 students was admitted by other means, e.g. by being members of the German armed forces or by the 8% quota for students from foreign countries. The mean age of the students was 21.3 ± 4.1 years. Their mean final high school grade was 1.52 ± 0.51 (1.0 is the best grade and 6.0 is the worst grade).The admission groups differed in their age (*F*(4291) = 128.26, *p* < .001). The students from the waiting period group and from the other reasons group were significantly older than all other groups and the students who gained their admission by a waiting period were significantly older than the students who were admitted by other reasons. The admission groups were also significantly different with respect to their grade point average (GPA) (*F*(4, 289) = 152.8, *p* < .001). Only the students who were admitted by HAM-Nat or by HAM-Int showed no significant difference in their GPA.

**Table 1:** Sociodemographic data of the participants

		High school degree	HAM-Nat	HAM-Int	Waiting period	Others
		(*N* = 50)	(*N* = 98)	(*N* = 66)	(*N* = 51)	(*N* = 33)
Age (years)		19.10 ± 1.40	19.40 ± 1.60	19.80 ± 1.90	28.10 ± 3.70^a^	22.60 ± 4.10^b^
Sex (F/M)		30/20	47/50	56/10	35/16	11/21
GPA		1.00 ± 0.00^c^	1.39 ± 0.19^d^	1.35 ± 0.19^e^	2.33 ± 0.39	1.79 ± 0.55^f^
BFI-10						
	*Extraversion*	3.50 ± 1.04	3.42 ± 0.99	3.65 ± 0.94	3.48 ± 1.01	3.48 ± 1.02
	*Agreeableness*	3.19 ± 0.78	3.60 ± 0.76^g,h^	3.40 ± 0.82	3.34 ± 0.73	3.14 ± 0.73
	*Conscientious.*	4.05 ± 0.80^i,j^	3.60 ± 0.76	3.77 ± 0.75	3.58 ± 0.79	3.67 ± 0.79
	*Neuroticism*	2.94 ± 0.99	3.01 ± 0.83	3.05 ± 0.9	2.91 ± 0.92	2.70 ± 0.98
	*Openness*	3.41 ± 0.75	3.34 ± 0.75	3.30 ± 0.76	3.39 ± 0.74	3.24 ± 0.67
GSE		30.22 ± 3.74	29.89 ± 3.42	28.77 ± 3.46	29.49 ± 4.58	29.54 ± 4.21
PHQ-9		4.35 ± 3.49	5.24 ± 4.02	4.20 ± 3.53	4.59 ± 2.97	5.67 ± 4.37
GAD-7		3.57 ± 3.73	4.02 ± 3.35	3.97 ± 3.41	3.47 ± 2.57	4.60 ± 2.99

Notes: *GPA* grade point average, *BFI-10* Big Five Inventory, *GSE* General self-efficacy scale, *PHQ-9* patient health questionnaire 9, *GAD-7* generalized anxiety disorder 7. For age, GPA, BFI-10), GSE, PHQ-9 and GAD-7 mean and SD are reported. For sex (F: female, M: male), the sample size (N) is reported. a = Waiting period vs. High school degree, HAM-Nat, HAM-Int and Others: *p* < .001; b = Others vs. High school degree, HAM-Nat and HAM-Int: *p* < .001; c = High school degree vs. HAM-Nat, HAM-Int, Waiting period and Others: *p* < .001; d = HAM-Nat vs. Waiting period and Others: *p* < .001; e = HAM-Int vs. waiting period and Others: *p* < .001; f = Others vs. Waiting period: *p* < .001; g = Ham-Nat vs. High school: *p* = .03; h = Ham-Nat vs. others: *p* = .04; i = High school degree vs. Ham-Nat: *p* = .04; j = High school degree vs. waiting period: *p* = .02

At the beginning of their first year, 2.7% of our sample showed a moderate and 0.7% a major level of depression. Five percent had a moderate and 2% a major level of anxiety. There were no significant differences between the admission groups in the GAD-7 (*F*(4, 291) = 0.29, *p* = .89) and in the PHQ-9 (*F*(4, 291) = 1.44, *p* = .22) scores. Our sample reached a mean of 29.59 ± 3.8 in the GSE and there were no significant differences of the standardized GSE between the different admission groups (*F*(4, 291) = 0.79, *p* = .53). A significant main effect occurred in the dimension Agreeableness (*F*(4, 291) = 2.15, *p* = .01) and Conscientiousness (*F*(4, 292) = 3.04, *p* = .02). Students who received their admission by the HAM-Nat had higher scores in Agreeableness than students who were admitted by their high school degree (*p* = .03) or other reasons (*p* = .04). Students who gained entrance to medical school by their high school degree had higher scores in Conscientiousness than students who were admitted by the HAM-Nat (*p* = .04) or by a waiting period (*p* = .02).

When the data of the MPS-H of our sample were transformed into t-scores, they showed a mean of 50.2 ± 10.13 in the SOP dimension. In the OOP dimension, the mean was 42.79 ± 10.63. The mean of the SPP dimension was 38.71 ± 10.42, indicating that our population is below one standard deviation of the mean of the normal population in this dimension. When the different medical school admission groups were compared (Table 2), the perfectionism scores showed a significant main effect in the SOP dimension (*F*(4, 288) = 3.52, *p* = .01; *f* = 0.06). Students who gained entrance to medical school by their high school degree had a significantly higher SOP score than students who gained entrance by a waiting period (*p* = .01). The groups also differed significantly in the OOP dimension (*F*(4, 288) = 3.32, *p* = .01; *f* = 0.1). Medical students who gained admission to medical school by other reasons had significantly higher scores in the OOP dimension than students who received their admission by HAM-Nat (*p* = .02), HAM-Int (*p* = .03) or by a waiting period (*p* = .01). In the MPS-F, only the dimension Personal Standard showed a significant main effect (*F*(4, 292) = 5.7, *p <* .001; *f* = 1.03). Students who gained entrance to medical school by their high school degree and by HAM-Nat had a significantly higher Personal Standard score than students who were admitted by a waiting period (*p* < .001 and *p = .01*, respectively). Students who were admitted by their high school degree showed significantly higher scores for Adaptive Perfectionism (AP) than students admitted by a waiting period (p < .001). Furthermore students who admitted by their HAM-Nat result had significantly higher scores than the students who admitted by their waiting period (*p* = .004).

**Table 2:** Analysis of variance of the group differences in the dimensions of perfectionism

Group						ANOVA		
	High school degree	HAM-Nat	HAM-Int	Waiting period	Others	*F*	h^2^	d.f.
	(M ± SD)	(M ± SD)	(M ± SD)	(M ± SD)	(M ± SD)			
MPS-H								
*SOP*	53.55 ± 10.03^a^	51.24 ± 9.91	49.48 ± 9.24	46.54 ± 10.99	49.12 ± 9.69	3.52*	.05	(4, 288)
*OOP*	42.61 ± 9.40	42.17 ± 11.63	42.14 ± 10.60	41.04 ± 9.12	49.00 ± 9.98^b,c^	3.32*	.04	(4, 288)
*SPP*	39.12 ± 11.95	38.60 ± 10.10	36.32 ± 9.82	40.54 ± 9.79	40.50 ± 9.04	1.52	.02	(4, 288)
MPS-F								
*CM*	24.18 ± 7.13	23.18 ± 7.11	21.28 ± 6.97	21.70 ± 7.55	21.00 ± 7.46	1.88	.03	(4, 292)
*PS*	30.67 ± 4.17^d^	29.25 ± 5.39	28.73 ± 4.72	26.29 ± 4.88	27.69 ± 6.39	5.70*	.07	(4, 292)
*PE*	12.68 ± 5.26	12.62 ± 4.38	11.73 ± 4.97	11.72 ± 3.45	12.42 ± 5.88	0.62	.01	(4, 293)
*PC*	6.88 ± 3.42	7.45 ± 3.09	6.89 ± 3.33	7.80 ± 3.52	7.88 ± 4.19	0.98	.01	(4, 293)
*DA*	10.68 ± 3.57	11.04 ± 3.09	11.01 ± 3.55	10.84 ± 3.13	10.88 ± 4.33	0.11	.00	(4, 291)
*O*	29.00 ± 4.84	27.20 ± 5.21	27.48 ± 4.66	27.92 ± 4.75	27.79 ± 6.39	1.10	.02	(4, 291)
CM								
*AP*	0.68 ± 1.66^e^	0.24 ± 1.83^f^	- 0.06 ± 1.67	- 0.86 ± 1.76	- 0.26 ± 1.82	5.56**	.07	(4, 289)
*MP*	0.22 ± 2.40	0.21 ± 2.25	- 0.33 ± 2.50	- 0.19 ± 2.47	- 0.06 ± 2.90	0.65	.01	(4, 288)

Notes: *MPS-H* multidimensional perfectionism scale by Hewitt and Flett, *SOP* self-oriented perfectionism, *OOP* other-oriented perfectionism, *SPP* socially prescribed perfectionism, *MPS-F* multidimensional perfectionism scale by Frost et al., *CM* concern over mistakes, *PS* personal standards, *PE* parental expectations, *PC* parental criticism, *DA* doubts about action, *O* organization, *AP* adaptive perfectionism, *MP* maladaptive perfectionism, *d.f.* degrees of freedom. The reported MPS-F scores are transformed standardized t-scores. * = *p* < 0.05; ** = *p* < .001. a = High school degree vs. waiting period: *p* = .01; b = Others vs. HAM-Nat: *p* = .02; c = Others vs. HAM-Int: *p* = .03; d = High school degree vs. waiting period: *p* < .001; High school degree vs. waiting period: *p* < .001; f = HAM-Nat vs. waiting period: *p* = .004

The four variables (Maladaptive Perfectionism, Agreeableness, Parental Criticism and Organization) which were included in the stepwise regression analysis explain 22% of the variance (*f* ^2^ = .28) for the prediction of the PHQ-9 score (Table 3). Regarding the GAD-7 score, Maladaptive Perfectionism and Neuroticism were included and explain 20% of the variance (*f* ^*2*^ = .25).

**Table 3:** Regression analysis for predicting PHQ-9 and GAD-7 scores

	beta	t	*p*	*R*∆^*2*^	*F*	*df1*	df*2*	*p*
PHQ								
				.22	20.32	4	275	.000
MP	.35	5.88	.000					
Agreeableness	−.13	−2.43	.016					
PC	.13	2.19	0.30					
O	−.11	−2.13	.034					
GAD-7								
				.20	35.76	2	278	.000
MP	.34	5.80	.000					
Neuroticism	.19	3.29	.001					

Notes: *MP* maladaptive perfectionism, *PC* parental criticism, *O* organization, *df* degrees of freedom. For the stepwise regression analysis the following variables were used: Sex, age, GPA, GSE, SOP, OOP, SPP, Personal Standards, Concern over mistakes, Parental Expectations, Parental Criticism, Doubts about Action, Organization, Adaptive Perfectionism, Maladaptive Perfectionism, Neuroticism, Extraversion, Openness, Conscientiousness and Agreeableness were used. Variables with a significance level of .05 were included while variables with a significance level of at least .10 were excluded

## Discussion

The medical students from our cohort revealed significantly lower scores in Socially-Prescribed Perfectionism (SPP) than the general population regardless of their way of admission to medical school. Low scores for SPP have been described previously for health professional students [8]. Since high scores for SPP in medical students have been shown to be negatively correlated with academic self-efficacy, which can ultimately trigger academic burnout [33], we hypothesize that low SPP scores might be a desired personality trait to look for in the admission process of medical students.

Medical students who were selected for medical school by their high school degree showed the highest score for Adaptive Perfectionism (AP), consisting of significantly higher scores for Self-Oriented Perfectionism (SOP) and Personal Standards compared with students who were admitted by a waiting period. Students admitted by their high school degree also revealed significantly higher scores for Conscientiousness in the BFI-10 than the students who were selected by a waiting period. In university students, Rice et al. found a significant association between the subscale High Standards of the Almost Perfect Scale-Revised (APS-R) [34] and the Five-Factor Model of Personality dimension of Conscientiousness [35], which underscores our finding for the subgroup of students selected for medical school by their high school degree. Conscientiousness, which includes self-achievement and self-discipline, has shown to significantly predict final scores in medical students’ pre-clinical years [36] and premedical students with higher perfectionistic strivings show better academic performance than students with lower levels of perfectionism [37]. The finding that academic achievements of medical students measured by grades and cognitive tests correlated with conscientiousness, an important aspect of clinical performance, was already published in 1979 [38]. Additionally, Conscientiousness predicts longitudinal increases in SOP in adolescents [39], which may also be an important finding with respect to medical students’ performance. Recently, it has been shown that applicants with distinct personality profiles and different attitudes towards the physicians’ roles in the society can be selected by combining a personality questionnaire with a single interview [40].

Interestingly, academic grade point average was associated with the overall performance of Australian medical students in all years of undergraduate medical training, while admission to medical school by a national cognitive aptitude test was associated with better performance in the pre-clinical years and admission by an interview assessing non-academic qualities was associated with better performance in the clinical years [41]. In our study, students who were admitted by the natural sciences test HAM-Nat showed the highest scores for Agreeableness in the BFI-10. Furthermore, students who were more agreeable showed less symptoms of depression. Agreeableness has demonstrated to be a personality trait associated with the acceptance to enter medical school after participation in multiple mini-interviews [42]. Students in our study, who were admitted to medical school by other reasons, e.g. being members of the armed forces, showed a significantly higher score for Other-Oriented Perfectionism (OOP), the tendency to expect perfection from other people, as reflected by the sample item, “The people who matter to me should never let me down”. Whether the high score for OOP displays a military socialization characteristic with an internalized Pygmalion effect [43] and whether this might have an influence on performance during undergraduate medical education will need further investigation. Additionally, Maladaptive Perfectionism was the strongest predictor in our study for the occurrence of depression and anxiety symptoms regardless of the way of admission itself. To combine perfectionism questionnaires with other selection tests might help to select students for medical school who are less prone to depression and anxiety, which prevent students from successful studies and which impede physicians’ work. When students with increased risk of anxiety or depression symptoms are already admitted to medical school a mentoring program as it exists in our medical school [44], might decrease the threshold of students to seek individual support from their mentor who could help to initiate treatment. Furthermore, mentoring might also be a means to increase general self-efficacy of the students.

Although the response rate in our study is acceptable, the goal to include the complete cohort of first year medical students was not reached. Non-responders either were not present in Hamburg during the orientation week or submitted incomplete questionnaires. In addition, our study was only performed at one medical school, which hampers generalizability. Another limitation of our study is the use of BFI-10, which includes only two items for each dimension, making it less reliable. However, we chose the BFI-10 to reduce the total number of items the students had to answer, but it derogates our findings with respect to the Big Five. Furthermore, due to admission regulations for our medical school the number of students in the different groups is not equal and thus making comparisons more difficult. Despite these limitations and the additional limitation that different admission processes to medical school exist in other countries, our study shows that different ways of admission to medical schools are associated with different personality characteristics of perfectionism. Socially Prescribed Perfectionism is lower in all admission groups compared to the general population, which should be a requirement for medical school admission because working as a physician requires a lot of intrinsic motivation with respect to life-long learning. Only students admitted by their high school degree show a significantly higher score for Adaptive Perfectionism and conscientiousness, which has been shown to be positively associated with medical school achievements. Students with high scores for Maladaptive Perfectionism showed higher scores for depression and anxiety, which might impede their study success.

## Conclusions

Students from all admission groups should be observed longitudinally to assess whether students who gained admission by a waiting period perform less well during undergraduate studies and to monitor whether perfectionism scores change during medical training in the different admission groups. Personality traits such as perfectionism, which predict successful learning or good behavior as a physician might play a more prominent role in medical school admission in the future.

## Abbreviations

AP: Adaptive perfectionism
BFI-10: Big five inventory 10
CM: Concern over mistakes
DA: Doubts about action
GAD-7: Generalized anxiety disorder 7
GSE: General self-efficacy scale (GSE)
MP: Maladaptive perfectionism
MPS-F: Multidimensional perfectionism scale by Frost
MPS-H: Multidimensional perfectionism scale by Hewitt and Flett
O: Organization
OOP: Other-oriented perfectionism
PC: Parental criticism
PE: Parental expectations
PHQ-9: Patient health questionnaire 9 (PHQ-9)
PS: Personal standards (PS)
SOP: Self-oriented perfectionism
SPP: Socially-prescribed perfectionism (SPP)

## References

[1] Frost RO, Marten P, Lahart C, Rosenblate R (1990). The dimensions of perfectionism. Cognit Ther Res.

[2] Flett GL, Hewitt PL, Flett GL, Hewitt PL (2002). Perfectionism. Perfectionism: theory and research.

[3] Loxterkamp D (2013). What do you expect from a doctor? Six habits for healthier patient encounters. Ann Fam Med.

[4] Humphris G, Kaney S (1998). The encouragement of “perfect” health professionals. Med Educ.

[5] Enns MW, Cox BJ, Sareen J, Freeman P (2001). Adaptive and maladaptive perfectionism in medical students: a longitudinal investigation. Med Educ.

[6] Wallace JE, Lemaire JB, Ghali WA (2009). Physician wellness: a missing quality indicator. Lancet.

[7] Compton MT, Carrera J, Frank E (2008). Stress and depressive symptoms/dysphoria among US medical students results from a large, nationally representative survey. J Nerv Ment Dis.

[8] Henning K, Ey S, Shaw D (1998). Perfectionism, the imposter phenomenon and psychological adjustment in medical, dental, nursing and pharmacy students. Med Educ.

[9] Leinster S (2013). Selecting the right medical student. BMC Med.

[10] Cohen-Schotanus J, Muijtjens AM, Reinders JJ, Agsteribbe J, van Rossum HJ, van der Vleuten CP (2006). The predictive validity of grade point average scores in a partial lottery medical admission system. Med Educ.

[11] Poole P, Shulruf B, Rudland J, Wilkinson T (2012). Comparison of UMAT scores and GPA in prediction of performance in medical school: a national study. Med Educ.

[12] Leddy JJ, Moineau G, Puddester D, Wood TJ, Humphrey-Murto S (2011). Does an emotional intelligence test correlate with traditional measures used to determine medical school admission?. Acad Med.

[13] Basco WT, Lancaster CJ, Gilbert GE, Carey ME, Blue AV (2008). Medical school application interview score has limited predictive validity for performance on a fourth year clinical practice examination. Adv Health Sci Educ Theory Pract.

[14] Knorr M, Hissbach J (2014). Mulitple mini-interviews: same concept, different approaches. Med Educ.

[15] Lievens F (2013). Adjusting medical school admission: assessing interpersonal skills using situational judgement tests. Med Educ.

[16] Schripsema NR, van Trigt AM, Borleffs JC, Cohen-Schotanus J (2014). Selection and study performance: comparing three admission processes within one medical school. Med Educ.

[17] Hubbeling D (2017). Lottery for medical school admission. Med Teach..

[18] McManus IC, Dewberry C, NIcholson S, Dowell JS, Woolf K, Potts HW (2013). Construct-level predictive validity of educational attainment and intellectual aptitude tests in medical student selection: meta regression of six UK longitudinal studies. BMC Med.

[19] Hissbach J, Klusmann D, Hampe W (2011). Dimensionality and predictive validity of the HAM-Nat, a test of natural sciences for medical school admission. BMC Med Educ.

[20] Hissbach JC, Sehner S, Harendza S, Hampe W (2014). Cutting costs of multiple mini-interviews - changes in reliability and efficiency of the Hamburg medical school admission test between two applications. BMC Med Educ..

[21] Schripsema NR, van Trigt AM, van der Wal MA, Cohne-Schotanus J (2016). How different medical school selection processes call upon different personality characteristics. PLoS One.

[22] Rammstedt B, John OP (2007). Measuring personality in one minute or less: a 10-item short version of the big five inventory in English and German. J Res Pers.

[23] Schwarzer R, Jerusalem M, Weinmann J, Wright S, Johnston M (1995). Generalized self-efficacy scale. Measures in health psychology: a user’s portfolio causal and control beliefs.

[24] Kroenke K, Spitzer RL, Williams JBW (2001). The PHQ-9: validity of a brief depression severity measure. J Gen Intern Med.

[25] Spitzer R, Kroenke K, Williams J, Löwe B (2006). A brief measure for assessing generalized anxiety disorder. Arch Inern Med.

[26] Hewitt PL, Flett GL (2004). Multidimensional perfectionism scale (MPS): technical manual.

[27] Stöber J. Skalendokumentation "Persönliche Ziele von SchülerInnen". In: Dalbert C, editor. Hallesche Berichte zur Pädagogischen Psychologie Nr. 3. Halle (Saale). Germany: Institut für Pädagogik, Martin-Luther-Universität Halle-Wittemberg; 2002. p. 1–50.

[28] Altstötter-Gleich C, Bergemann N (2006). Testgüte Einer Deutschsprachigen Version der Mehrdimensionalen Perfektionismus Skala von Frost, Marten, Lahart und Rosenblate (MPS-F). Diagnostica.

[29] Rammstedt B, Kemper CJ, Beierlein C, Kovaleva A (2012). Eine kurze Skala zur Messung der fünf Dimensionen der Persönlichkeit: Big-Five-Inventory-10 (BFI-10). GESIS Work Pap.

[30] Schwarzer R, Jerusalem M. (Hrsg.) Skalen zur Erfassung von Lehrer- und Schülermerkmalen. Dokumentation der psychometrischen Verfahren im Rahmen der Wissenschaftlichen Begleitung des Modellversuchs Selbstwirksame Schulen. Freie Universität Berlin, Berlin, 1999.

[31] Martin A, Rief W, Klaiberg A, Braehler E. Validity of the brief patient health questionnaire mood scale (PHQ-9) in the general population. Gen Hosp Psychiatry. 2006;28(1):71–7. 10.1016/j.genhosppsych.2005.07.003 16377369

[32] Löwe B, Müller S, Brähler E, Kroenke K, Albani C, Decker O. (2007). Validierung und Normierung eines kurzen Selbstratinginstrumentes zur Generalisierten Angst (GAD-7) in einer repräsentativen Stichprobe der deutschen Allgemeinbevölkerung. Psychother Psychosom Med Psychol. 2007;57(2):A050.

[33] JH Y, Chae SJ, Chang KH (2016). The relationship among self-efficacy, perfectionism and academic burnout in medical school students. Korean J Med Educ.

[34] Slaney RB, Rice KG, Mobley M, Trippi J, Ashby JS (2001). The revised almost perfect scale. Meas Eval Couns Dev.

[35] Rice KG, Ashby JS, Slaney RB (2007). Perfectionism and the five-factor model of personality. Assessment.

[36] Lievens F, Coetsier P, De Fruyt F, De Maeseneer J (2002). Medical students’ personality characteristics and academic performance: a five-factor model perspective. Med Educ.

[37] Sevlever M, Rice KG (2010). Perfectionism, depression, anxiety and academic performance in premedical students. CMEJ.

[38] Willoughby TL, Gammon LC, Jonas HS (1979). Correlates of clinical performance during medical school. J Med Educ.

[39] Stoeber J, Otto K, Dalbert C (2009). Perfectionism and the big five: conscientiousness predicts longitudinal increases in self-oriented perfectionism. Pers Individ Dif.

[40] Talmor AG, Falk A, Almog Y (2017). A new admission method may select applicants with a distinct personality profile. Med Teach.

[41] Sladek RM, Bond MJ, Frost LK, Prior KN (2016). Predicting success in medical school: a longitudinal study of common Australian student selection tools. BMC Med Educ.

[42] Jerant A, Griffin E, Rainwater J, Henderson M, Sousa F, Bertakis KD, Fenton JJ, Franks P (2012). Does applicant personality influence multiple mini-interview performance and medical school acceptance offers?. Acad Med.

[43] Eden D, Shani AB (1982). Pygmalion goes to boot camp: expectancy, leadership, and trainee performance. J Appl Psychol.

[44] Guse J, Schweigert E, Kulms G, Heinen I, Martens C, Guse AH (2016). Effects of mentoring speed dating as an innovative matching tool in undergraduate medical education: a mixed methods study. PLoS One.

